# Sleep, movement, and marks: exploring the relationship between sleep quality, physical activity, and academic performance in male university students

**DOI:** 10.7717/peerj.21154

**Published:** 2026-04-17

**Authors:** Mshari Alghadier

**Affiliations:** Department of Health and Rehabilitation Sciences, Prince Sattam bin Abdulaziz University, Kharj, Saudi Arabia

**Keywords:** Academic performance, Physical activity, Sleep quality, University students

## Abstract

**Background:**

Sleep quality and physical activity (PA) are modifiable behaviors that may influence the cognitive functioning and academic performance of university students. However, evidence focusing specifically on male students in Middle Eastern settings remains limited.

**Objective:**

To examine the associations between sleep quality, PA, and academic performance among male university students.

**Methods:**

In this cross-sectional study, 130 male undergraduates from Prince Sattam bin Abdulaziz University in Saudi Arabia completed a sociodemographic questionnaire, the Arabic Pittsburgh Sleep Quality Index (PSQI), and the Arabic International Physical Activity Questionnaire–Short Form (IPAQ-SF). Height and weight were measured to calculate body mass index (BMI). Academic performance was assessed *via* self-reported cumulative grade point average (GPA; 5-point scale). Sleep quality was classified as good (PSQI ≤ 5) or poor (PSQI > 5), and PA was categorized as low, moderate, or high according to IPAQ scoring guidelines. Group differences in GPA were examined *via* one-way ANOVA and independent-samples *t* tests. Pearson correlations and multiple linear regression were used to explore independent associations between sleep, PA, and GPA, adjusting for age and BMI.

**Results:**

The participants had a mean age of 21.7 ± 1.6 years and a mean BMI of 25.2 ± 6.1 kg/m^2^; 65.4% were classified as having poor sleep quality. The PA levels were approximately evenly distributed (low, 33.1%; moderate, 33.1%; high, 33.8%). The overall mean GPA was 3.01 ± 0.48, and it differed significantly across PA categories (F(2,127) = 83.55, *p* < 0.001, *ηp*^2^ = 0.57; low 2.57 ± 0.32, moderate 3.00 ± 0.31, high 3.45 ± 0.33) and between good and poor sleepers (t(86) = 4.98, *p* < 0.001; 3.28 ± 0.45 *vs* 2.87 ± 0.43, Cohen’s d = 0.93). In the regression analysis (*R*^2^ = 0.64, *p* < 0.001, f^2^ = 1.78), higher PA (B = 0.43, *p* < 0.001) and better sleep quality (lower PSQI; B = –0.04 per point, *p* < 0.001) independently predicted higher GPA, whereas BMI was not a significant predictor.

**Conclusion:**

Poor sleep quality is highly prevalent and, together with insufficient PA, is associated with lower academic performance among male university students. Despite the limitations of a cross-sectional, single-gender design, campus-based interventions that promote both sleep hygiene and regular PA may contribute to improved academic outcomes in this population.

## Introduction

University students are exposed to multiple academic, social, and environmental pressures that can adversely affect their lifestyle behaviors, including sleep and physical activity (PA). Both sleep and movement are central to brain health, emotional regulation, and cognitive performance and therefore may have direct implications for academic achievement. Experimental and observational studies consistently show that inadequate or dysregulated sleep impairs attention, working memory, executive function, and learning consolidation—domains that are critical for success in higher education. Systematic reviews of university populations indicate that a broad range of sleep problems (short duration, poor quality, irregular schedules, insomnia symptoms, and excessive daytime sleepiness) are associated with poorer academic performance, although effect sizes and methodological quality vary (Suardiaz-Muro et al., 2020; Gardani et al., 2022; Barley, Godinho & Scullin, 2025).

Across regions, poor sleep quality is highly prevalent in university students. A recent meta-analysis of African universities reported that more than half of students experience poor sleep quality and highlighted consistent associations with mental distress and suboptimal academic outcomes (Nakie et al., 2024). In Saudi Arabia and neighboring countries, cross-sectional studies among medical and health sciences students have likewise documented very high rates of poor sleep quality and insomnia, often exceeding 70–80%, with evidence that poorer sleepers tend to report lower grade point averages (GPA) and greater academic difficulties (Elsayed et al., 2022; Khaled et al., 2025). Population-based data from Saudi Arabia further suggest that poor sleep quality and insomnia are common in the general adult population, underscoring a broader contextual pattern of sleep disturbance that likely extends to university campuses (Senitan, BinDhim & Althumiri, 2025; Baklola et al., 2024; Thalib et al., 2024).

In parallel, PA has emerged as a key determinant of cognitive and academic outcomes. Regular PA is associated with improved cerebral blood flow, neurotrophic factor expression, mood, and stress regulation, which may translate into better concentration, information processing, and academic performance (Redondo-Flórez, Ramos-Campo & Clemente-Suárez, 2022; Hillman, Erickson & Kramer, 2008; Castelli et al., 2007; Maass et al., 2015; Szuhany, Bugatti & Otto, 2015; Salmon, 2001). At the population level, the World Health Organization estimates that roughly one-third of adults worldwide do not meet recommended PA levels and that physical inactivity continues to rise in many countries, despite clear health and economic consequences (World Health Organization, 2022; Strain et al., 2024). University students are not exempt from this trend; several studies indicate that a substantial proportion of students are insufficiently active, particularly as they progress through demanding academic programs (Dinger, Behrens & Han, 2006).

Empirical findings in higher education settings suggest that PA is positively associated with academic performance, although the magnitude and consistency of associations vary by context and measurement approach. A recent systematic review and meta-analysis concluded that physical exercise has a moderate positive effect on academic performance among university students internationally (Rosales-Ricardo & Cáceres-Manzano, 2024). Correlational and cohort studies in health-professional and medical students have reported that those who meet PA guidelines or participate in higher volumes of moderate-to-vigorous PA are more likely to attain higher GPAs or pass critical examinations (Redondo-Flórez, Ramos-Campo & Clemente-Suárez, 2022; Raja et al., 2025; Li & Zhang, 2022). In Saudi universities, emerging data from health colleges also support a positive correlation between PA levels and academic achievement, suggesting that the promotion of PA may have academic as well as health benefits in this population (Mohsen Aljuaid & Saeed Aljulaymi, 2024).

Despite growing evidence for independent links between sleep, PA, and grades, relatively few studies have examined these behaviors concurrently within the same models, which limits the understanding of their relative and combined contributions to academic performance. Many available studies focus on either sleep or PA in isolation, rely on self-reported grades, and often do not adjust for potential confounders such as age, body mass index (BMI), smoking, or year of study (Barley, Godinho & Scullin, 2025; Rosales-Ricardo & Cáceres-Manzano, 2024). In addition, much of the literature pools male and female students, even though gender norms, daily schedules, and activity opportunities differ substantially in many societies, including those with gender-segregated campuses. As a result, data specific to male university students—particularly in Middle Eastern settings—remain limited, and it is unclear whether associations reported in mixed-gender or predominantly female samples generalize to male-only cohorts (Elsayed et al., 2022; Khaled et al., 2025).

Robust measurements of sleep and PA are essential to clarify these relationships. The Pittsburgh Sleep Quality Index (PSQI) is one of the most widely used instruments for assessing subjective sleep quality and disturbances over the preceding month. It has demonstrated acceptable reliability and validity across diverse university student samples, including male students in India and Nigeria and college populations in other low- and middle-income regions, supporting its use for large-scale epidemiological studies (Manzar et al., 2015; Aloba et al., 2007; De Moraes et al., 2024; Smyth, 1999). The International Physical Activity Questionnaire–Short Form (IPAQ-SF) is similarly recommended as a brief, cost-effective tool for estimating weekly PA in adults and has shown acceptable test–retest reliability and comparable validity to other self-reported PA instruments in college students and adults (Dinger, Behrens & Han, 2006; Lee et al., 2011; Craig et al., 2017; Helou et al., 2017; Ács et al., 2021). Together, these instruments enable parallel characterization of sleep and PA in large student samples and facilitate comparisons with international literature.

Therefore, studies that simultaneously examine sleep quality and PA as correlates of academic performance in male university students, use validated instruments and account for key confounders, are needed (Craig et al., 2017; Jahrami et al., 2019; Dewald et al., 2010). Such evidence could inform campus-based health promotion initiatives targeting sleep hygiene and PA as modifiable levers to support academic success, particularly in contexts where male students face high academic demands and may underutilize available health services (Galdas, Cheater & Marshall, 2005). This population is unique because male students in Saudi Arabia often navigate a social environment characterized by late-night activities and family obligations that significantly shift sleep-wake cycles. Analyzing sleep and PA concurrently is essential because they are behaviorally reciprocal; however, most regional literature treats them as isolated variables, which may lead to an incomplete understanding of their joint contribution to academic success (BaHammam et al., 2011; Alshammari et al., 2023; Dolezal et al., 2017).

Thus, the present cross-sectional study aims to examine the associations between sleep quality, PA, and academic performance among male undergraduate students in a university setting. Specifically, we seek (1) to describe the prevalence of poor sleep quality and insufficient PA in this population; (2) to explore bivariate associations between sleep quality, PA levels, and GPA; and (3) to determine the independent contributions of sleep quality and PA to academic performance after adjusting for sociodemographic and health-related covariates. We hypothesize that poorer sleep quality and lower PA levels are associated with lower GPA and that sleep quality, in particular, remains an independent predictor of academic performance after controlling for PA and other confounders.

## Methods

### Study design and participants

This cross-sectional study was conducted during the 2025 academic year at the College of Applied Medical Sciences, Prince Sattam bin Abdulaziz University, Saudi Arabia. Male undergraduate students enrolled in the College of Applied Medical Sciences (560 students) were invited to participate *via* convenience sampling. Participants were primarily enrolled in health-related programs, including physical therapy, radiology, biomedical technology, and medical laboratory sciences, across all four undergraduate year levels. The inclusion criteria were as follows: (a) male sex; (b) aged 18–25 years; (c) full-time enrollment in an undergraduate program; and (d) ability to read and understand Arabic. The exclusion criteria were as follows: (a) self-reported diagnosis of major neurological, psychiatric, or sleep disorders (*e.g.*, epilepsy, major depression, obstructive sleep apnea); (b) known severe chronic disease (*e.g.*, cancer, advanced cardiac or renal disease) likely to markedly affect sleep or PA; and (c) current use of medications known to substantially affect sleep (*e.g.*, sedative–hypnotics).

A total of 130 male students met the eligibility criteria and provided complete data on sleep quality, PA, GPA, and covariates; these participants formed the final analytic sample. No a priori sample size calculation was conducted; however, the achieved sample size exceeded common rules of thumb (≥10–15 participants per predictor) for multiple linear regression with four predictors.

### Data collection procedure

Participants were recruited through multiple channels, including invitation emails sent *via* university mailing lists, social media posts on official student platforms, and advertisement posters displayed in common areas of the college. Each announcement briefly described the study aims and eligibility criteria and directed interested students to contact the researcher. Students who expressed interest were provided with an information sheet explaining the study in detail and were asked to provide written informed consent prior to participation.

Data collection took place in the Motion Capture Laboratory at the Health and Rehabilitation Sciences Department. The participants completed Arabic self-report questionnaires (sociodemographic form, Arabic PSQI, Arabic IPAQ short form) under the supervision of the researcher, who was available to clarify any procedural questions. Immediately after questionnaire completion, body weight and height were measured *via* standardized procedures. For academic performance, the students reported their GPA for each of the previous four semesters (5-point scale) listed in the university electronic portal, and these values were averaged to calculate a mean GPA for analysis. A course instructor cross-checked the reported GPA values against the portal to confirm their accuracy. All the data were then deidentified *via* numeric codes prior to analysis to maintain participant anonymity.

### Tools and instruments

#### (1) Sociodemographic and academic variables

A brief sociodemographic form captured age (years), year of study, major, smoking status (yes/no), and handedness. Academic performance was operationalized as self-reported cumulative GPA on the university’s 5-point scale (range 1.0–5.0), recorded to two decimal places. Self-reported GPA has been shown to be highly correlated with official records in university samples and is widely used in academic performance research (Kuncel, Credé & Thomas, 2005).

#### (2) Anthropometric measurements

Body weight was measured to the nearest 0.1 kg *via* a calibrated digital weighing scale with participants wearing light clothing and no shoes. Height was measured to the nearest 0.5 cm using a portable stadiometer, with participants standing upright, heels together, and head in the Frankfurt plane. BMI was calculated as weight in kilograms divided by height in meters squared (kg/m^2^). Participants were categorized as underweight (BMI < 18.5 kg/m^2^), healthy weight (18.5–24.9 kg/m^2^), overweight (25.0–29.9 kg/m^2^), or obese (≥30.0 kg/m^2^) according to the World Health Organization criteria (WHO Consultation on Obesity (1999: Geneva, Switzerland) & World Health Organization, 2000).

#### (3) Sleep quality: Arabic Pittsburgh Sleep Quality Index

Subjective sleep quality over the preceding month was assessed *via* the Arabic version of the PSQI. The original PSQI is a 19-item self-report measure yielding seven component scores (subjective sleep quality, sleep latency, sleep duration, habitual sleep efficiency, sleep disturbances, use of sleep medication, and daytime dysfunction), which are summed to produce a global score ranging from 0-21; higher scores indicate poorer sleep quality. A global score >5 is conventionally used to classify “poor sleepers” (Buysse et al., 1989).

The Arabic PSQI used in this study is based on the translation and psychometric evaluation described by Suleiman et al. (2010), who followed forward–backward translation procedures and demonstrated acceptable internal consistency (Cronbach’s α = 0.77) and construct validity in Arabic-speaking adults. Subsequent studies have supported the reliability and validity of Arabic PSQI versions in diverse Arabic-speaking populations, including patients with cancer and medical students, with Cronbach’s *α* values typically ≥ 0.70 and satisfactory test–retest reliability (Al Maqbali et al., 2020; Alqarni et al., 2025). In the present sample, the PSQI was administered in Modern Standard Arabic; a global score >5 was used to classify poor sleep quality, which is consistent with prior literature (Suleiman et al., 2010; Al Maqbali et al., 2020; Alqarni et al., 2025). For analysis, we used the PSQI global score as a continuous variable and dichotomized participants into “good sleep” (PSQI ≤ 5) and “poor sleep” (PSQI >5) categories. The component scores were retained for descriptive and exploratory analyses.

#### (4) Physical activity: Arabic International Physical Activity Questionnaire–Short Form

Habitual PA over the previous seven days was assessed *via* the Arabic version of the IPAQ-SF. The IPAQ-SF comprises seven items capturing the frequency and duration of walking, moderate-intensity activity, vigorous-intensity activity, and sitting time in the last week. Scores are typically converted to metabolic equivalent (MET)-minutes per week for walking, moderate, vigorous, and total PA using standard scoring protocols from the IPAQ committee (Craig et al., 2017). In this study, we used an Arabic translation of the IPAQ-SF that follows recommended translation and cultural adaptation guidelines (forward–backward translation, expert panel review, and pilot testing). Arabic versions of the IPAQ (long and short forms) have demonstrated acceptable reliability and validity in Arabic-speaking adult populations, including Lebanese adults (Helou et al., 2017) and Saudi or Gulf-region samples, where self-reported Arabic IPAQ measures have shown reasonable agreement with objective devices (*e.g.*, accelerometers, activity monitors) and satisfactory test–retest reliability (Al Ozairi et al., 2024; Garashi et al., 2020; Arumugam et al., 2024).

Following standard IPAQ scoring guidelines, total weekly PA (MET-min/week) was calculated by summing the products of reported minutes and days in each intensity category multiplied by the corresponding MET value (3.3 METs for walking, 4.0 METs for moderate activity, and 8.0 METs for vigorous activity). The participants were then categorized into three PA levels—low, moderate, and high—using the established cutoff points of the IPAQ for health-enhancing PA (Craig et al., 2017). For some analyses, the IPAQ category was also coded ordinally (Low = 1, Moderate = 2, High = 3) to reflect ordered increases in activity.

### Statistical analysis

All analyses were conducted *via* R Software (R version 2023.6.01, R Foundation for Statistical Computing, Vienna, Austria). The data were first examined for completeness and plausibility; out-of-range values and internal inconsistencies were checked against the original questionnaires. No variable exceeded 5% missingness; therefore, a complete-case analysis was used.

Continuous variables (*e.g.*, age, BMI, PSQI global score, GPA) are summarized as the means and standard deviations (M ± SD) and ranges; categorical variables (*e.g.*, BMI category, IPAQ level, sleep quality category) are summarized as counts and percentages. The normality of the GPA and other key continuous variables was inspected *via* histograms, Q–Q plots, and Kolmogorov–Smirnov tests; the GPA was approximately normally distributed, permitting the use of parametric tests.

Between-group differences in GPA across IPAQ categories (low, moderate, high) were assessed *via* one-way analysis of variance (ANOVA). When the overall F test was significant, Bonferroni-adjusted pairwise comparisons were conducted to identify differences between specific activity levels. Differences in GPA between good and poor sleepers were examined *via* independent-samples *t* tests (Welch’s correction was used if the homogeneity of variances was violated, as indicated by Levene’s test). Effect sizes were reported as Cohen’s d for *t* tests and *η*^2^ (or partial *η*^2^) for ANOVA.

Associations between continuous variables (GPA, PSQI global score, IPAQ ordinal score, age, BMI) were examined *via* Pearson product–moment correlation coefficients. A correlation heatmap was constructed to visually summarize the magnitude and direction of these associations. To examine the independent contributions of sleep quality and PA to academic performance, a multiple linear regression model was fitted with GPA as the dependent variable and the IPAQ ordinal score, PSQI global score, age, and BMI as predictors. Regression assumptions (linearity, normality and homoscedasticity of residuals, absence of multicollinearity) were checked by inspecting residual plots, probability plots, and variance inflation factors (VIFs). Unstandardized coefficients (B), standard errors (SE), 95% confidence intervals (CI), t statistics, and *p* values are reported. Model fit was evaluated *via* the coefficient of determination (R^2^ and adjusted R^2^) and the overall F statistic.

In addition, model-based effect plots were generated to illustrate the adjusted relationship between the PSQI global score and the predicted GPA across the observed range of sleep quality, stratified by PA level, with age and BMI held at their sample means. These plots were produced in R *via* the predicted values and 95% CI derived from the regression model. All tests were two-tailed, and a *p* value <0.05 was considered statistically significant.

#### Ethical considerations

The study received ethical approval from the Departmental Research Ethics Committee at Prince Sattam bin Abdulaziz University, Saudi Arabia (No. RHPT/025/014; date: 15/09/2025). All procedures complied with the principles of the Declaration of Helsinki. Written informed consent was obtained from the participants. The confidentiality and anonymity of the participant data were maintained throughout the study.

## Results

Descriptive analysis (Table 1) revealed that more than 40% of the participants fell outside the healthy weight range, with a substantial proportion classified as overweight or obese. Notably, poor sleep quality was highly prevalent in this cohort, affecting nearly two-thirds of the students. Physical activity levels were approximately evenly distributed across the low, moderate, and high categories. The cohort’s academic performance, measured by cumulative GPA, showed considerable variation across the sample, ranging from 2.00 to 4.11 on a 5-point scale.

**Table 1:** Participant characteristics (*n* = 130).

**Variable**	**Mean ± SD**	**Range**	**n (%)**
**Age (years)**	21.68 ± 1.56	19–25	–
**Weight (kg)**	76.06 ± 18.52	40–120	–
**Height (cm)**	171.77 ± 7.56	155–190	–
**BMI (kg/m^2^)**	25.21 ± 6.12	14.51–41.52	–
**BMI category**			
−** Underweight**	–	–	17 (13.1%)
−**Healthy weight**	–	–	54 (41.5%)
−**Overweight**	–	–	25 (19.2%)
−**Obese**	–	–	34 (26.2%)
**PSQI global score**	6.42 ± 2.44	2–12	–
**Sleep quality category**			
−** Good sleep (PSQI ≤ 5)**	–	–	45 (34.6%)
−** Poor sleep (PSQI > 5)**	–	–	85 (65.4%)
**Physical activity (IPAQ category)**			
−** Low**	–	–	43 (33.1%)
−** Moderate**	–	–	43 (33.1%)
−** High**	–	–	44 (33.8%)
**GPA (5-point scale)**	3.01 ± 0.48	2.00–4.11	–

**Notes.**

BMI body mass index PSQI Pittsburgh Sleep Quality Index IPAQ International Physical Activity Questionnaire GPA grade point average kg kilogram cm centimeter

**Figure 1:**
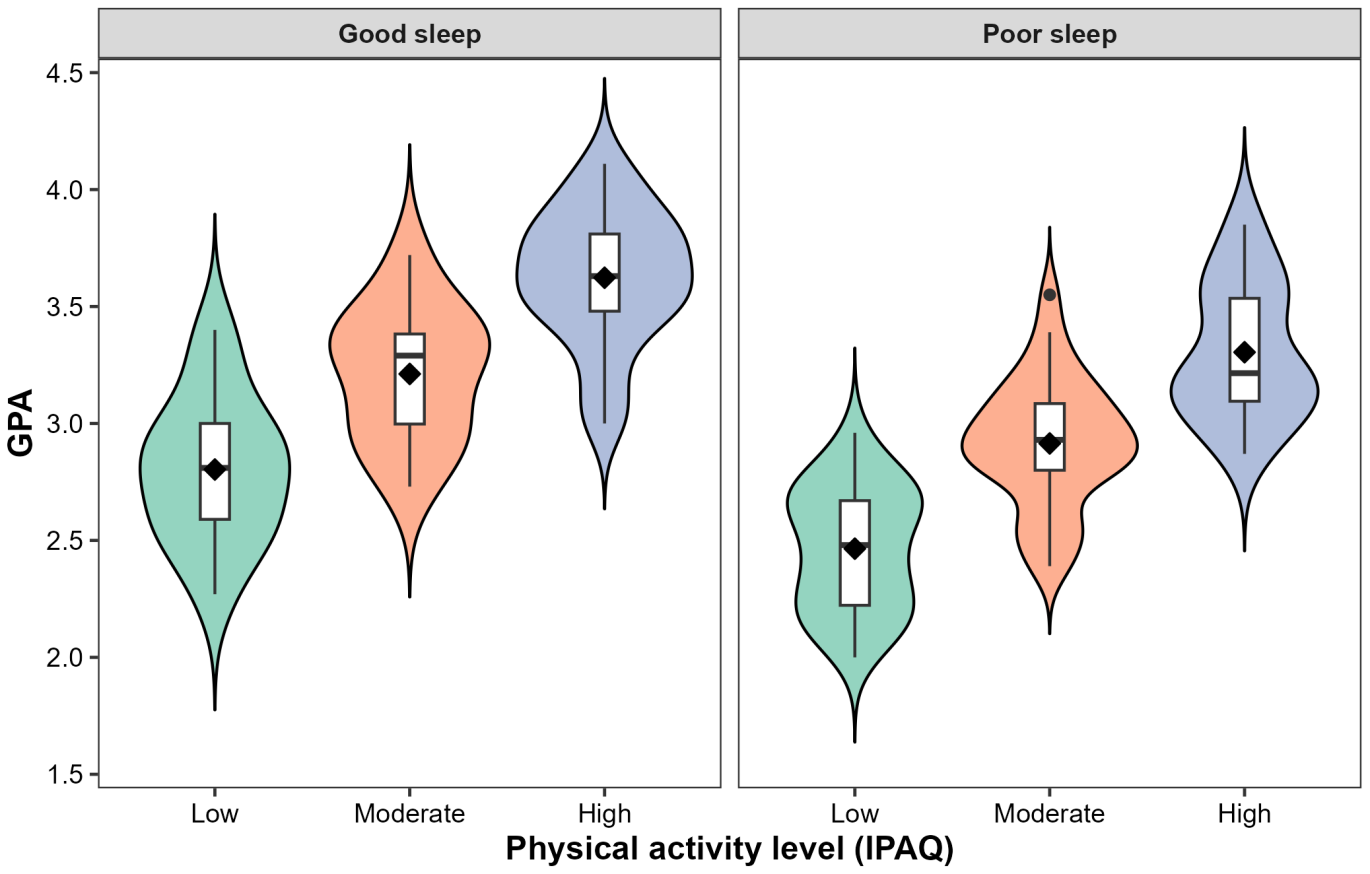
The distribution of the grade point average (GPA) across physical activity levels (low, moderate, high) stratified by sleep quality (good sleep *vs* poor sleep). Each violin depicts the full distribution of GPA within each group, with overlaid boxplots (median and interquartile range) and mean markers. Higher GPA values cluster among students with high physical activity and good sleep quality, whereas lower GPA values concentrate among students with low physical activity and poor sleep quality.

GPA differed markedly across PA categories and sleep quality groups. Students with high PA had the highest mean GPA (3.45 ± 0.33), followed by those with moderate PA (3.00 ± 0.31), whereas students with low PA had the lowest mean GPA (2.57 ± 0.32). One-way ANOVA revealed a significant main effect of IPAQ category on GPA (F(2,127) = 83.55, *p* < 0.001, *ηp*^2^ = 0.57. Bonferroni-adjusted *post hoc* comparisons indicated that students in the high PA group achieved significantly higher GPAs than those in both the moderate and low activity groups, and the moderate group significantly outperformed the low activity group (all *p* < 0.001). Detailed pairwise comparisons and effect sizes are provided in Table S1. With respect to sleep, good sleepers presented a greater GPA (3.28 ± 0.45) than poor sleepers did (2.87 ± 0.43); this difference was significant according to an independent-samples t test (t(86) = 4.98, *p* < 0.001, Cohen’s d = 0.93). The joint distribution of GPA by PA and sleep quality is illustrated in Fig. 1.

**Figure 2:**
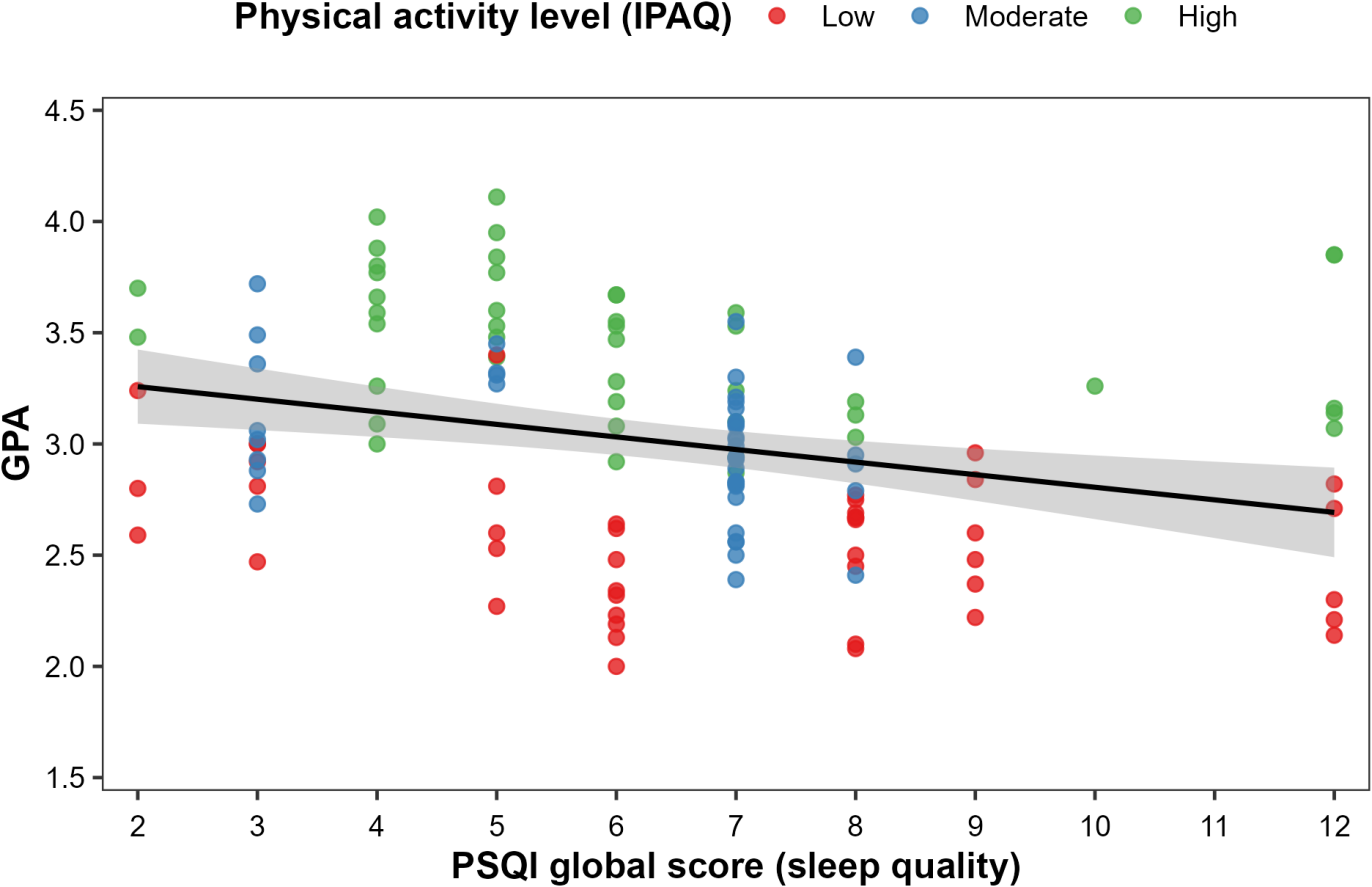
The association between sleep quality and academic performance in male university students. Points represent individual participants, with PSQI global scores on the *x*-axis (higher scores indicate poorer sleep) and GPA on the *y*-axis, colored by physical activity level (IPAQ category: low, moderate, high). The solid line represents the fitted linear regression line with a 95% confidence band for the overall sample. Poorer sleep quality is associated with lower GPA, and at any given PSQI level, students with higher physical activity tend to have higher GPA than those with lower activity.

Bivariate correlations corroborated these patterns. When the IPAQ score was coded ordinally (low = 1, moderate = 2, high = 3), greater PA was strongly and positively associated with GPA (*r* = 0.75, *p* < 0.001). In contrast, higher PSQI global scores (worse sleep) were moderately and negatively associated with GPA (*r* = −0.29, *p* < 0.001). Figure 2 displays the scatterplot of the PSQI global score *versus* GPA, with points colored by IPAQ category. A clear negative linear trend is evident, with higher PSQI scores associated with lower GPA. The figure also suggests that, at any given PSQI level, students with high PA tend to cluster toward higher GPAs than those reporting low PA does, indicating that PA may partially offset the adverse association between poor sleep and academic performance.

The correlation table (Table 2) summarizes the interrelationships among GPA, the PSQI global score, the IPAQ (ordinal score), age, and BMI. The PSQI global score was weakly positively correlated with BMI and age, suggesting that older and heavier students tended to report slightly poorer sleep, although these associations were small in magnitude. The correlations between BMI and GPA and between age and GPA were low, indicating that body composition and age played a relatively minor role in explaining academic performance compared with PA and sleep quality.

**Table 2:** Pearson correlations between GPA, sleep quality, physical activity, age, and BMI (*n* = 130).

**Variable**	**GPA**	**PSQI global**	**Age**	**BMI**
**PSQI global**	–0.29^***^			
**Age (years)**	–0.16	–0.17		
**BMI (kg/m^2^)**	–0.03	–0.18^*^	0.21^*^	
**IPAQ score**	0.75^***^	–0.10	–0.07	–0.16

**Notes.**

The values are Pearson’s r values.

GPA grade point average PSQI Pittsburgh Sleep Quality Index BMI body mass index

IPAQ score coded as low = 1, moderate = 2, and high = 3.

Significance level = * *p* < 0.05, ** *p* < 0.01, *** *p* < 0.001.

To examine the independent contributions of PA and sleep quality to academic performance, a multiple linear regression model was fitted with GPA as the dependent variable and IPAQ level (ordinal), PSQI global score, age, and BMI as predictors. The overall model was statistically significant and explained a substantial 64% of the variance in GPA. After adjusting for age and BMI, higher PA levels remained a robust independent predictor of better academic performance. Conversely, poorer sleep quality—indicated by higher PSQI scores—independently predicted lower GPA. While age showed a modest negative association with academic outcomes, BMI did not significantly contribute to the model after accounting for lifestyle behaviors. Detailed model parameters and coefficients are presented in Table 3.

**Table 3:** Multiple linear regression predicts GPA from physical activity, sleep quality, age, and BMI.

**Predictor**	**B**	**SE B**	**95% CI for B**	***t***	***p***
**Intercept**	3.38	0.40	[2.59, 4.18]	8.38	<0.001
**Physical activity (IPAQ score)**	0.43	0.03	[0.36, 0.49]	13.31	<0.001
**PSQI global score**	–0.04	0.01	[–0.07, –0.02]	–4.06	<0.001
**Age (years)**	–0.05	0.02	[–0.09, –0.02]	–2.98	0.004
**BMI (kg/m^2^)**	0.01	0.00	[–0.00, 0.02]	1.40	0.163
**Model summary**	*R*^2^ = 0.64, F(4, 125) = 55.61, *p* < 0.001, *f*^2^= 1.78				

**Notes.**

Dependent variable: GPA (5-point scale). Physical activity (IPAQ score) was coded as low = 1, moderate = 2, and high = 3.

BMI body mass index PSQI Pittsburgh Sleep Quality Index IPAQ International Physical Activity Questionnaire GPA grade point average

**Figure 3:**
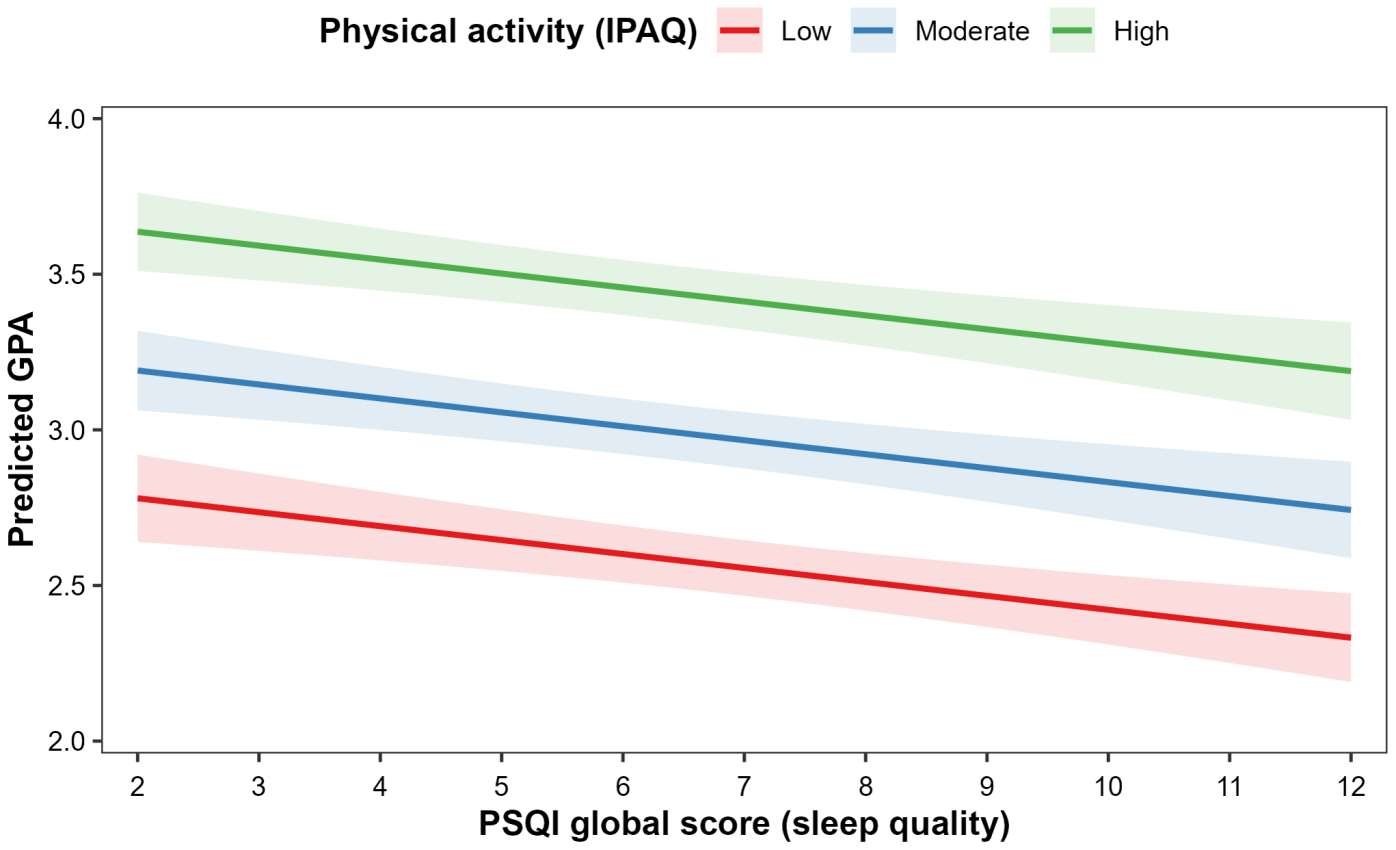
Model-based effect of sleep quality on predicted GPA, stratified by physical activity level. Lines represent adjusted predicted GPA across the observed range of PSQI global scores, derived from the multiple linear regression model including the IPAQ score, PSQI global score, age, and BMI. Age and BMI are held at their sample means, with separate curves for low, moderate , and high physical activity. The shaded areas denote 95% confidence intervals. The predicted GPA decreases as sleep quality worsens, and at any given PSQI value, students with higher physical activity are predicted to achieve higher GPAs than those with lower activity.

Figure 3 presents the model-based effect of sleep quality on the predicted GPA, stratified by PA level. The curves depict adjusted predicted GPA across the observed range of PSQI global scores, with age and BMI held at their mean values. Across all IPAQ categories, the predicted GPA decreases as the PSQI score worsens, confirming the inverse association between sleep quality and academic performance. The lines for higher PA levels are consistently shifted upward, indicating that, at any given level of the PSQI, students with higher PA are predicted to achieve better GPA than their less active peers. The confidence bands around the prediction lines are narrow, particularly in the central PSQI range, supporting the precision of the estimated effects. Together, these findings indicate that both insufficient PA and poor sleep quality are common in this cohort and exert independent, additive adverse effects on academic performance in male university students.

## Discussion

The present study examined how sleep quality and PA are related to academic performance in a sample of male university students in Saudi Arabia. Three main findings emerged. First, poor sleep quality was highly prevalent, with nearly two-thirds of the students classified as poor sleepers. Second, both better sleep quality and higher PA levels were associated with higher GPA, with PA showing a particularly strong positive association. Third, after adjusting for age and BMI, sleep quality and PA remained independent predictors of GPA, whereas BMI was not significantly related to academic performance.

### Sleep quality and academic performance

These findings are broadly consistent with a growing literature documenting links between sleep and academic outcomes in university students. A systematic review of 30 studies concluded that inadequate sleep—across multiple dimensions such as sleep duration, quality, and regularity—is generally associated with poorer academic performance (Suardiaz-Muro et al., 2020). More recently, Okano et al. (2019) demonstrated in a longitudinal study of 100 college students that both sleep duration and consistency significantly predict academic performance, with students sleeping less than 6 h showing markedly lower exam scores. Similarly, a comprehensive review of sleep and academic performance reported that more than 60% of studies reported at least one significant association between sleep variables and academic achievement (Barley, Godinho & Scullin, 2025). Our observation that poorer global sleep quality was moderately and negatively associated with GPA aligns with cross-sectional work in North American students, showing that poor PSQI-defined sleep quality is associated with lower GPA, even after accounting for depressive symptoms (Gilbert & Weaver, 2010; Gaultney, 2010).

Our finding that 65.4% of poor sleepers is notably higher than rates reported in some Western contexts. For example, Lund et al. (2010) reported that 60% of U.S. college students had poor sleep quality, whereas Becker et al. (2018) reported that 50.2% of a German university sample had poor sleep quality. However, our prevalence is comparable to that reported in recent studies in Asian contexts: Zhang, Peters & Chen (2018) reported 68.3% poor sleep quality among Chinese medical students, and Alsaggaf et al. (2016) reported 62.8% poor sleep quality among Saudi medical students. This suggests that sleep disturbances may be particularly pronounced in Middle Eastern and Asian university populations, potentially reflecting cultural factors such as later bedtimes, extended family obligations, and academic pressure (BaHammam et al., 2012; Cheng et al., 2012).

The present results also echo those of regional studies from Saudi Arabia and the wider Middle East. Among medical students at Alfaisal University in Riyadh, poor sleep quality was highly prevalent and associated with lower GPA and greater psychological distress (Alnasser et al., 2022). A study at Imam Abdulrahman bin Faisal University similarly reported that poorer PSQI scores and shorter sleep durations were linked to lower cumulative GPA in health sciences students (Hassan et al., 2025). Other Saudi cohorts, including medical students at King Saud University and Jazan University, have documented high rates of poor sleep quality *via* the PSQI, often exceeding 60%, with suggestive or significant associations with academic performance and daytime sleepiness (Al Shammari et al., 2020; Mahfouz et al., 2013).

The observed association between poor sleep quality and lower GPA is supported by robust mechanistic evidence. Sleep restriction and fragmented sleep have been shown to impair attention, working memory, executive functioning, and information consolidation—all of which are critical for effective study and exam performance (Killgore, 2010; Lim & Dinges, 2010). Experimental studies have demonstrated that even partial sleep deprivation (*i.e.,* sleep restriction < 6 h) for one week results in cumulative deficits in cognitive performance equivalent to up to two nights of total sleep deprivation (Van Dongen et al., 2003). Poor sleep may affect academic performance through multiple pathways. Direct effects include impaired attention during lectures, reduced information encoding, and compromised retrieval during examinations (Dewald et al., 2010). Indirect pathways include increased psychological distress, mood disturbance, and daytime fatigue, which reduce class attendance, study efficiency, and academic motivation (Hershner & Chervin, 2014; Levenson et al., 2016). A previous study revealed that poor sleep quality mediates the relationship between academic stress and depressive symptoms, suggesting a bidirectional cycle in which academic pressure disrupts sleep, which in turn impairs coping capacity (Alqudah et al., 2019).

### Physical activity as a robust predictor

Our findings regarding PA extend prior evidence suggesting that more active students tend to perform better academically. A recent meta-analysis of university students revealed that higher total PA was associated with better academic performance, although the authors rated the evidence as having low credibility and noted mixed findings across individual studies (Trott et al., 2024). Broader meta-analytic work across age groups reports small-to-moderate positive associations between PA and academic outcomes (Donnelly et al., 2016; Alvarez-Bueno et al., 2017). Within Saudi Arabia, studies at various universities have reported positive associations between PA and academic performance, although with varying effect sizes. A study at King Saud University revealed that students with high PA levels had significantly higher GPAs than did those with low PA levels (Al-Drees et al., 2016). Similarly, Mohsen Aljuaid & Saeed Aljulaymi (2024) reported that physically active health college students at King Khalid University achieved better educational outcomes.

However, not all evidence supports a positive PA–academic performance relationship, as some studies report no benefit of objectively measured PA or increased physical education time on academic outcomes, possibly due to differences in PA measurement, population characteristics, or socioeconomic confounding (Esteban-Cornejo et al., 2014; Käll, Nilsson & Lindén, 2014). The observed independent association between PA and GPA appeared notably robust in our cohort. While this relationship is stronger than those often reported in international literature, it should be interpreted with caution given the study’s specific context (Singh et al., 2012; Fedewa & Ahn, 2011). This magnitude might potentially reflect unique socio-cultural factors in Saudi Arabia, where high PA levels may serve as a proxy for superior self-regulatory skills in an environment where sedentary behaviors are common (Zayed et al., 2024; Bajuaifer & Alrashdi, 2025). Consequently, regular movement could possibly act as a neurocognitive buffer against academic pressures, though further longitudinal research is necessary to confirm such a link (Voss et al., 2013; Puterman et al., 2010).

This positive association may involve several neurobiological and psychosocial pathways. Physiologically, regular PA is thought to increase brain-derived neurotrophic factor expression, which may support synaptic plasticity and neurogenesis in regions vital for memory. Additionally, enhanced cerebral blood flow and oxygenation could potentially improve executive functions such as attention (Maass et al., 2015; Szuhany, Bugatti & Otto, 2015; Vaynman, Ying & Gomez-Pinilla, 2004). Beyond biological factors, PA may indirectly support academic outcomes by improving mood regulation or reducing psychological distress (Biddle et al., 2019; Rebar et al., 2015). In a university setting, engagement in PA might also be associated with improved time management and academic motivation (Hillman, Erickson & Kramer, 2008; Voss et al., 2013; Erickson et al., 2011).

### Joint influence and additive effects

Our findings contribute to the limited research examining the joint influence of sleep and PA on academic outcomes, as many previous studies have focused on these behaviors in isolation (Suardiaz-Muro et al., 2020; Trott et al., 2024). In our multivariable model, both higher PA and better sleep quality independently predicted higher GPA after adjusting for age and BMI. The effect plot showed approximately parallel declines in predicted GPA as the sleep quality worsened across all activity levels, suggesting additive rather than synergistic effects. This additive nature implies clear academic gradients, with physically active students who sleep well showing the highest predicted GPA (approximately 3.5) and those with low activity and poor sleep the lowest (approximately 2.5), which is consistent with models in which multiple lifestyle risks cumulatively affect performance and wellbeing (Poitras et al., 2016; Prochaska, Spring & Nigg, 2008).

Although these physiological pathways were not directly measured in the current study, such neurobiological mechanisms may be relevant to the observed findings. For instance, the significant GPA deficit between poor and good sleepers could potentially reflect impaired hippocampal-dependent memory consolidation, which is often compromised when PSQI scores exceed clinical thresholds. Similarly, the stepwise GPA increase across PA levels might reflect a dose–response relationship between movement and executive function maintenance under academic pressure. While not explicitly tested here, these biological pathways could be among the primary drivers behind the 64% variance in GPA explained by our model. Consequently, while interventions targeting either behavior may offer benefits, combined strategies addressing both sleep and PA appear likely to be most effective (Kredlow et al., 2015).

### BMI and public health implications

In contrast, BMI and age showed weaker associations with GPA. BMI was not a significant predictor in the multivariable model, and its correlations with GPA and sleep quality were small. This finding suggests that in this relatively young male group, lifestyle behaviors are more potent determinants of cognitive success than body composition alone. It is possible that the academic impact of obesity reported in other literatures is mediated by the sleep disturbances and physical inactivity we controlled for, indicating that health-promotion efforts should prioritize behavioral modification over weight-loss targets to support academic achievement (Taras & Potts-Datema, 2005; Datar & Sturm, 2006).

A meta-analysis of 38 studies reported a small negative association between BMI and academic performance with substantial heterogeneity (Santana et al., 2017), and adolescent data suggest that obesity-related stigma, rather than BMI itself, may underlie this link (Crosnoe & Muller, 2004). However, among university students, BMI was not significantly associated with GPA after controlling for other health behaviors (MacCann et al., 2020). Despite established links between obesity, sleep-disordered breathing, and daytime functioning (Jennum & Riha, 2009), our findings suggest that in this cohort, behavioral factors such as sleep and PA are more important for academic performance than body composition is. Furthermore, the crude nature of BMI—which does not distinguish fat from muscle—may further obscure associations with PA and fitness in physically active students (Burkhauser & Cawley, 2008).

### Regional physical activity trends and national health priorities

The high prevalence of insufficient PA in Saudi university students has been repeatedly documented and represents a significant public health concern. Our finding that approximately one-third of the male students were classified as having low activity aligns with broader regional data. Among university students specifically, studies from Riyadh, Jazan, Makkah, and Eastern Province reported that 40–60% fail to meet recommended PA levels (Mohsen Aljuaid & Saeed Aljulaymi, 2024; Al-Zalabani, Al-Hamdan & Saeed, 2015; Awadalla et al., 2014; Alkhateeb et al., 2019). Saudi students appear less active than their international peers: only approximately one-third of Saudi adolescents meet PA recommendations *versus* 60–80% in many European countries (World Health Organization, 2011), and among university students, an estimated 30–35% of Saudis meet PA guidelines compared with 40–50% in U.S. samples (Keating et al., 2005). Our finding that the low-activity group had the lowest mean GPA compared with the moderate- and high-activity groups reinforces concerns that physical inactivity is both a public health and an academic issue in this context. This dose−response relationship suggests that interventions promoting PA may yield dual benefits for health and educational attainment (Donnelly et al., 2016; Fornaguera, Dowda & Pate, 2024).

In addition to its academic implications, this study aligns closely with Saudi Arabia’s Vision 2030 agenda and its associated transformation programs (Kingdom of Saudi Arabia, 2025; Alasiri & Mohammed, 2022). Vision 2030 and the Health Sector Transformation Program explicitly emphasize the prevention of health risks, the promotion of healthy lifestyles, and the restructuring of the health system to support a “Vibrant Society” (Alharthi, Alharthi & Alharthi, 2019; Ministry of Health, 2025). In parallel, the Quality of Life Program and the Saudi Sports for All Federation target substantial increases in community participation in PA—from 13% in 2015 to 40% by 2030—as a means to reduce the burden of noncommunicable disease (NCD) and increase human capital (Quality of Life Program, 2018; Saudi Sports for All Federation, 2019). National reports on NCDs in Saudi Arabia highlight insufficient PA and other lifestyle factors as key, modifiable drivers of morbidity and economic loss and call for early, population-level interventions among youth and young adults (Alqunaibet et al., 2021). By documenting high rates of poor sleep quality and suboptimal PA alongside their associations with GPA in male university students, our findings provide locally relevant evidence on a critical segment of the future workforce. These data can inform campus-based and digital health initiatives designed to promote active lifestyles and healthy sleep within universities, thereby complementing Vision 2030’s broader goals of improving population health, educational attainment, and long-term productivity.

### Strengths, limitations, and future directions

Several methodological strengths support the interpretation of our findings. We used validated Arabic versions of the PSQI and IPAQ-SF, instruments that have demonstrated acceptable reliability and construct validity in Arabic-speaking populations, including Saudi students and young adults (Suleiman et al., 2010; Al Maqbali et al., 2020; Alqarni et al., 2025; Arumugam et al., 2024). Sleep and PA were analyzed both categorically and continuously, and we adjusted for key covariates (age and BMI) in the regression model. Moreover, our visualizations (violin plots, scatterplots, and effect plots) provide converging evidence for the statistical findings and aid in interpreting the magnitude and direction of associations.

However, several limitations should be acknowledged. First, the cross-sectional design precludes causal inference. While it is plausible that poor sleep and low PA contribute to poorer academic performance, reverse causation (*e.g.*, academic stress leading to reduced sleep and exercise) and bidirectional relationships are also possible. Longitudinal and intervention studies are needed to clarify temporal ordering and causality (Rosales-Ricardo & Cáceres-Manzano, 2024; Babaeer et al., 2022; Wunsch et al., 2021). Second, all behavioral variables were self-reported, which introduces potential recall and social desirability bias. Objective measures such as actigraphy or accelerometry, combined with sleep diaries, would provide a more precise characterization of sleep–activity patterns (Ndupu et al., 2023; Cowdell & Dyson, 2019). Third, the sample was drawn from a single university and included only male students, limiting its generalizability to female students, other institutions, and different cultural contexts. Gender differences in sleep patterns, PA, and academic stress have been described in university populations and warrant explicit investigation.

Despite these caveats, the present findings have practical implications. Universities in Saudi Arabia and similar settings may wish to integrate sleep education and PA promotion into student support services and campus health programs. Interventions combining sleep hygiene counseling, time management training, and accessible opportunities for moderate-to-vigorous PA (*e.g.*, structured exercise classes, active campus design) could be tested for their impact on both well-being and academic outcomes. From a policy perspective, aligning academic timetables, campus recreation facilities, and digital health tools to support healthy sleep–activity routines may help address the high burden of poor sleep and physical inactivity observed among university students in the region (Almutairi et al., 2018; Hershner & O’Brien, 2018; Chase & Conn, 2013).

Future research should build on this work by employing longitudinal designs, recruiting gender-balanced and multi-institutional samples, and incorporating objective measures of sleep and PA. It would also be valuable to examine additional mediators and moderators—such as mental health, screen time, and academic stress—to better understand how sleep and activity translate into academic performance. By situating our findings within a rapidly expanding international and regional evidence base, this study underscores that improving sleep quality and PA is not only a health priority but also a potentially important strategy for enhancing academic success among male university students in Saudi Arabia.

## Supplemental Information

10.7717/peerj.21154/supp-1 Supplemental Information 1 Raw data

10.7717/peerj.21154/supp-2 Supplemental Information 2 Codebook

10.7717/peerj.21154/supp-3 Supplemental Information 3 Pairwise Comparisons of GPA Across Physical Activity Levels (Bonferroni Post Hoc Test) Mean GPA values: Low (2.57 ± 0.32), Moderate (3.00 ±0.31), and High (3.45 ± 0.33).

10.7717/peerj.21154/supp-4 Supplemental Information 4 STROBE checklist

10.7717/peerj.21154/supp-5 Supplemental Information 5 Evidence for open access IPAQ

10.7717/peerj.21154/supp-6 Supplemental Information 6 Evidence for open acces PSQI

## Additional Information and Declarations

### Data Availability

The following information was supplied regarding data availability: The raw data is available in the Supplemental File and at FigShare: Alghadier, Mshari (2025). Sleep and PA. figshare. Dataset. https://doi.org/10.6084/m9.figshare.30747566.v1
