# Supplementary material for: Sleep, movement, and marks: exploring the relationship between sleep quality, physical activity, and academic performance in male university students
Source: PeerJ. 2026 Apr 17;14:e21154. doi: 10.7717/peerj.21154 (PMC13094551; doi:10.7717/peerj.21154)
Supplement: Supplemental Information 2 [file peerj-14-21154-s002.pdf]

1. Dataset overview

- gender – text (all “Male”)
- age – numeric (years)
- weight – numeric (kg)
- height – numeric (cm)
- BMI – numeric (kg/m^2)
- BMI_cat – text (Underweight / Healthy weight / Overweight / Obese)
- Pref_hand – text (Right / Left)
- IPAQ level – text (Low / Moderate / High)
- PSQI_comp1–7 – numeric 0–3
- PSQI_Global Score – numeric 0–21
- PSQI_cat – text (Good sleep / Poor sleep)
- GPA – numeric (0–5 scale)

2. PSQI component codebook (0–3 factors)

PSQI_comp1: Subjective sleep quality

Code	Label
0	Very good
1	Fairly good
2	Fairly bad
3	Very bad

PSQI_comp2: Sleep latency

Code	Label
0	≤ 15 minutes
1	16–30 minutes
2	31–60 minutes
3	> 60 minutes

PSQI_comp3: Sleep duration

Code	Label
0	> 7 hours
1	6–7 hours
2	5–6 hours
3	< 5 hours

PSQI_comp4: Sleep efficiency

(Habitual sleep efficiency = [time asleep / time in bed] × 100)

Code	Label
0	≥ 85%
1	75–84%
2	65–74%
3	< 65%

PSQI_comp5: Sleep disturbance

Code	Label
0	No difficulty in the past month
1	< once a week
2	Once or twice a week
3	≥ three times a week

PSQI_comp6: Use of sleep medication

Code	Label
0	Not during the past month
1	Less than once a week
2	Once or twice a week
3	Three or more times a week

PSQI_comp7: Daytime dysfunction

Code	Label
0	No problem / not during the past month
1	Slight problem / < once a week
2	Moderate problem / once or twice a week
3	Severe problem / ≥ three times a week

3. PSQI_Global Score: PSQI_cat

PSQI_Global Score	PSQI_cat label
0–5	Good sleep
> 5	Poor sleep

4. BMI: BMI_cat

Using WHO BMI categories:

BMI (kg/m²)	BMI_cat label
< 18.5	Underweight
18.5–24.9	Healthy weight
25.0–29.9	Overweight
≥ 30.0	Obese

5. IPAQ level (categorical)

- Standard IPAQ categorical scoring (for reference; your file has IPAQ level as "Low/Moderate/High"):
- Low – does not meet criteria for Moderate or High.
- Moderate – e.g., ≥ 600 MET-minutes/week (some combination of walking/moderate/vigorous activity).
- High – e.g., ≥ 1500 MET-minutes/week vigorous on ≥ 3 days, or ≥ 3000 MET-minutes/week overall.
