# Supplementary material for: Sleep, movement, and marks: exploring the relationship between sleep quality, physical activity, and academic performance in male university students
Source: PeerJ. 2026 Apr 17;14:e21154. doi: 10.7717/peerj.21154 (PMC13094551; doi:10.7717/peerj.21154)
Supplement: Supplemental Information 3 — Mean GPA values: Low (2.57 ± 0.32), Moderate (3.00 ±0.31), and High (3.45 ± 0.33). [file peerj-14-21154-s003.docx]

**Supplementary Table 1. Pairwise Comparisons of GPA Across Physical Activity Levels (Bonferroni Post Hoc Test)**

| **Comparison (IPAQ Category)** | **Mean Difference** | **SE** | **95% Confidence Interval** | **p-value** | **Cohen’s d** |
| --- | --- | --- | --- | --- | --- |
| **High PA vs. Moderate PA** | 0.45 | 0.07 | [0.28, 0.62] | < 0.001 | 1.41 |
| **High PA vs. Low PA** | 0.88 | 0.07 | [0.71, 1.05] | < 0.001 | 2.71 |
| **Moderate PA vs. Low PA** | 0.43 | 0.07 | [0.26, 0.60] | < 0.001 | 1.36 |

*Mean GPA values: Low (2.57 ± 0.32), Moderate (3.00 ± 0.31), and High (3.45 ± 0.33).*
