# Supplementary material for: Sleep, movement, and marks: exploring the relationship between sleep quality, physical activity, and academic performance in male university students
Source: PeerJ. 2026 Apr 17;14:e21154. doi: 10.7717/peerj.21154 (PMC13094551; doi:10.7717/peerj.21154)
Supplement: Supplemental Information 5 [file peerj-14-21154-s005.pdf]

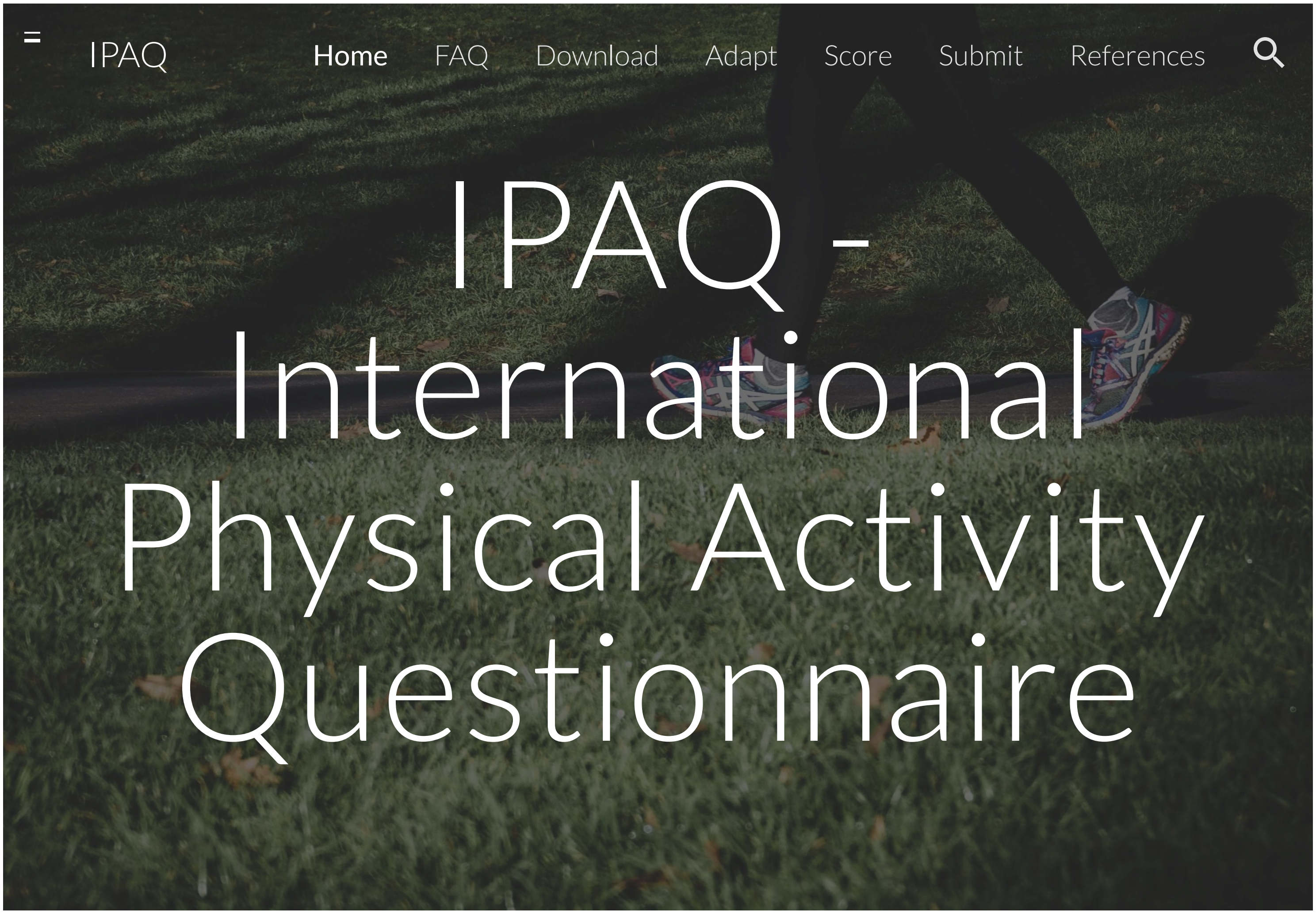

IPAQ - International Physical Activity Questionnaire

Welcome!

The International Physical Activity Questionnaire (IPAQ) is a well-developed and widely-used instrument that can be used to obtain comparable estimates of physical activity between populations and countries. It was designed for physical activity surveillance so it is particularly useful for large population studies, but can be used with care in other contexts.

On this site you will find:

- background about the development of the IPAQ questionnaire
- information about the correct use of the questionnaire
- information about how you can adapt the questionnaire
- links to the questionnaire itself, in multiple languages

Please note:

- The IPAQ questionnaire is **publicly available**, it is **open access**, and **no permissions are required to use it.**
- We unfortunately cannot provide individual support to researchers or students. This website is maintained on a purely voluntary basis.
- We strongly recommend you begin by reading the Frequently Asked Questions!

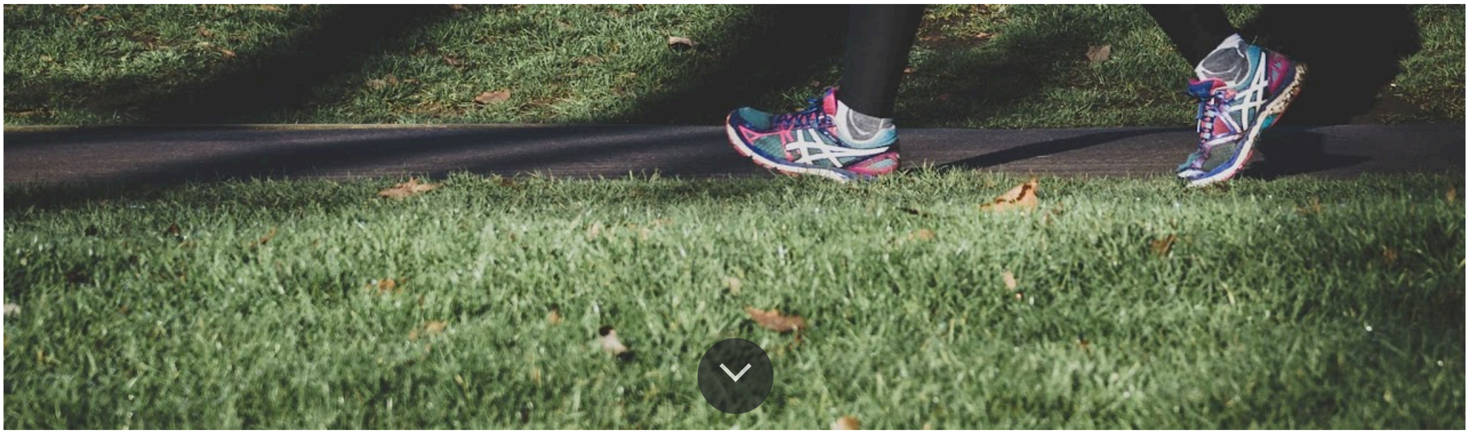

IPAQ 2022
