# Supplementary material for: Sleep, movement, and marks: exploring the relationship between sleep quality, physical activity, and academic performance in male university students
Source: PeerJ. 2026 Apr 17;14:e21154. doi: 10.7717/peerj.21154 (PMC13094551; doi:10.7717/peerj.21154)
Supplement: Supplemental Information 6 [file peerj-14-21154-s006.pdf]

University of Pittsburgh

Department of Psychiatry

The Center for Sleep and Circadian Science

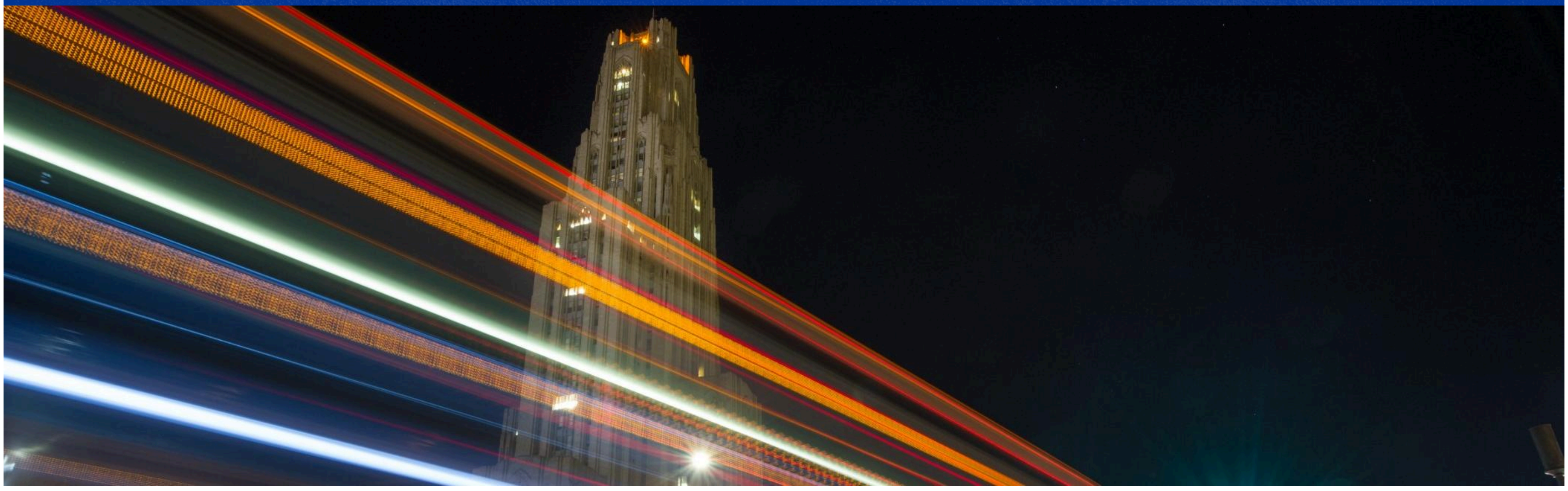

The Pittsburgh Sleep Quality Index (PSQI)

What is the PSQI?

The Pittsburgh Sleep Quality Index (PSQI) is a widely used self-report questionnaire that assesses sleep quality over a one-month time interval. The measure was developed by Dr. Daniel Buysse, Dr. Charles Reynolds, Dr. Timothy Monk, Dr. Susan Berman, and Dr. David Kupfer at the University of Pittsburgh. Since the PSQI's publication in 1989, it has been cited in over 34,000 peer-reviewed articles.

The PSQI is commonly used in both clinical and research settings to evaluate various aspects of sleep. It is a valuable tool for assessing sleep quality as it captures multiple dimensions of sleep, including both subjective experiences and objective parameters. It allows researchers and healthcare providers alike to obtain a comprehensive understanding of an individual's sleep patterns and disturbances and inform treatment decisions and interventions for sleep disorders.

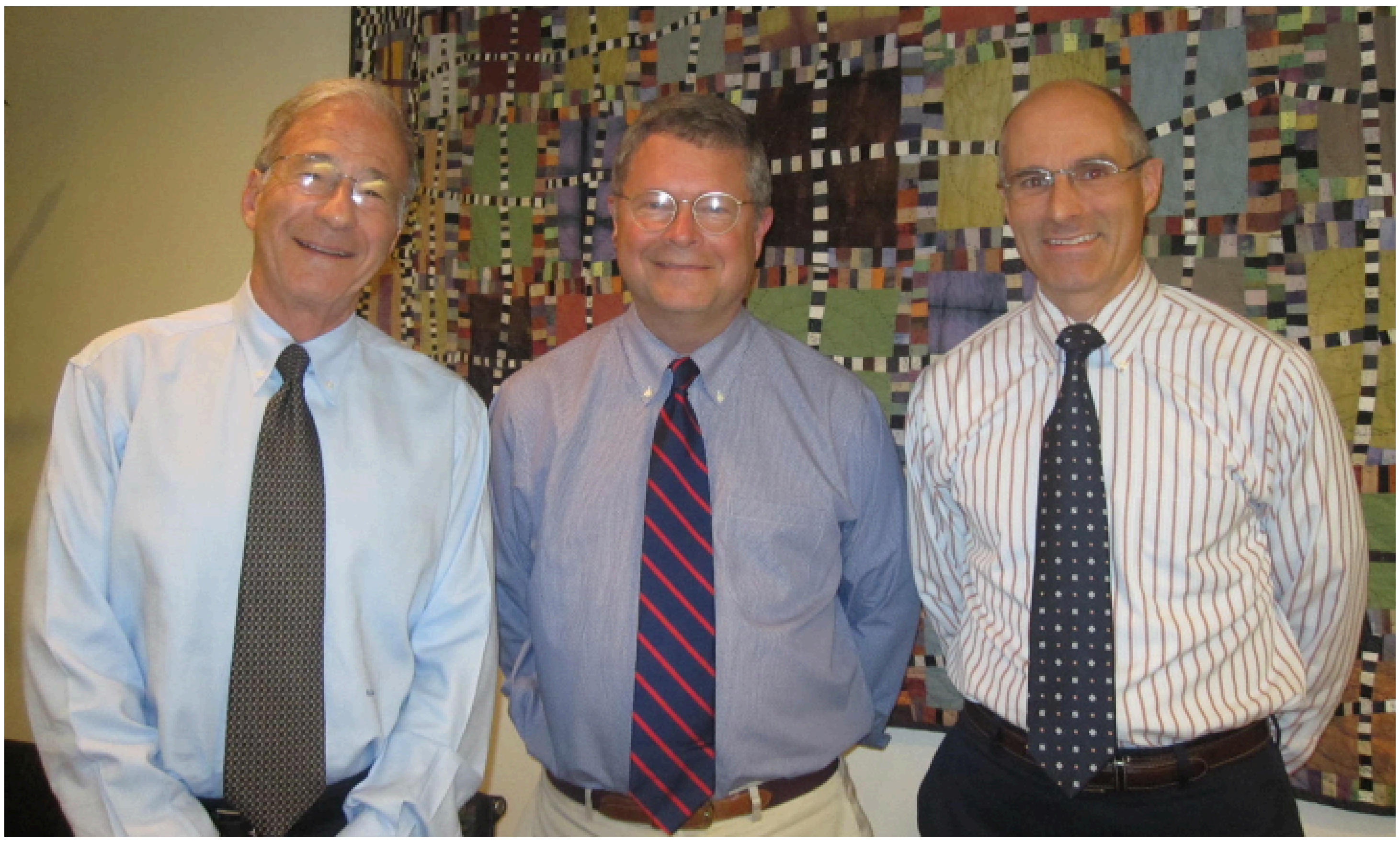

Three of the PSQI's developers (left to right): Dr. Kupfer, Dr. Reynolds, and Dr. Buysse

Validity of the PSQI

The validity of the Pittsburgh Sleep Quality Index (PSQI) was rigorously examined. Significant differences were observed in global PSQI scores between subject groups, with control subjects differing from patient groups. Further analysis demonstrated distinct profiles for different patient groups, reinforcing the PSQI's ability to discriminate between varying levels of sleep disturbances. Covariate analysis confirmed the robustness of these findings, with age and sex having minimal impact on group differences.

Additionally, the PSQI's validity was supported by its consistent performance in identifying individuals with sleep disturbances using a post hoc cutoff score. Comparative analysis with polysomnography further substantiated the PSQI's validity, showing alignment with objective measures of sleep quality across various subject groups.

Overall, these findings underscore the PSQI's reliability and utility in assessing sleep quality across diverse populations and contexts. More information on PSQI's validity can be found in the original article.

Using the PSQI

Please cite the reference for the PSQI in any publications:

Buysse,D.J., Reynolds,C.F., Monk,T.H., Berman,S.R., & Kupfer,D.J. (1989). The Pittsburgh Sleep Quality Index (PSQI): A new instrument for psychiatric research and practice. Psychiatry Research, 28(2), 193-213.

If you intend to reproduce or reprint the original PSQI article, please fill out the request form.

The PSQI instrument is owned by the University of Pittsburgh and may be reprinted without charge only for non-commercial research and educational purposes. You may not make changes or modifications of this form without prior written permission from the University of Pittsburgh. If you would like to use this instrument for commercial purposes or for commercially sponsored research, please fill out the request form.

The PSQI has been **translated** into 56 additional languages. For more information on these languages, or to request the PSQI in a different language, follow the link to the MAPI website. You will have to create a profile (free) and then click on PSQI Request for Translations under Conditions of Use heading.

Not sure where to start? Fill out the request form!

Scoring the PSQI

Each component score of the PSQI ranges from 0 to 3, with 3 indicating the greatest dysfunction or disturbance. The seven component scores are then summed to obtain a global PSQI score, which ranges from 0 to 21. Higher scores indicate poorer sleep quality, with a score greater than 5 suggesting significant sleep difficulties

Documents

- PSQI Original Article
- PSQI Instrument
- PSQI Scoring
- PSQI Scoring Database (PSQI Scoring Database.zip)
- How to Download and Use the PSQI Database
- PSQI Language Translation List

Interested in accessing the PSQI? Request Form

PSQI Price Sheet

**Research**		
	Use of PSQI in non-commercial study (academic or US government funded)	FREE
	Use of PSQI in commercial study (funded by for profit entity)	$20,000 per Study
**Publication**		
	Link to the English version of PSQI in an academic publication	FREE

	Include the full PSQI in an academic publication	$5,000
	Include the full PSQI in a commercial publication	Per use fee*
**Educational Program**	Link to the PSQI in an educational program	FREE
	Include the full PSQI in an academic educational program	$5,000
	Include the full PSQI in a commercial educational program	Per use fee*
**Clinical Use**	Use of the PSQI (paper version) in a single location	FREE
	Use of the PSQI in an Electronic Health Record	Per use fee*
**Other Uses**	Use of the PSQI for internal commercial use	Per use fee*
	Distribution of the PSQI or data generated from the use of the PSQI to customers/ clients	Varies/ Please contact

***Per Use Fee Structure (Annual)**

< 1,000 uses	$5,000
1,001 – 2,500 uses	$9,000
2,501 – 5,000 uses	$12,000
5,001 – 10,000 uses	$15,000
> 10,000 uses	$20,000

Validation Articles

- The PSQI: Validity and factor structure in young people
- Criterion validity of the PSQI: Investigation in a non-clinical sample
- Validation of a 3-factor scoring model for the PSQI in older adults
- The PSQI as a screening tool for sleep dysfunction in clinical and non-clinical samples: A systematic review and meta-analysis
- Brief version of the PSQI (B-PSQI) and measurement invariance across gender and age in a population-based sample
- Psychometric evaluation of the PSQI

PSQI FAQs

Is the PSQI reliable/valid?

Yes! For more information on the validity/reliability of the PSQI, please refer to the original PSQI article linked in Documents.

Are PSQI translations reliable/valid?

Yes! PSQI translations have received the Linguistic Validation Certificate. This confirms that the translations are...

- Conceptually equivalent to the original and comparable across languages;
- Culturally relevant to the context of the target country;
- Easily understood by the people to whom the translated instrument is administered.

Can I modify the assessment time frame?

- The PSQI is only validated for the time frame specified in the PSQI (monthly assessment). If you collect in a different time frame, the instrument will no longer be considered validated.

For more information on the linguistic validity of PSQI translations, email vlachosc@upmc.edu

Quick Links

- Training
- Measures and Instruments
